# Trends in Syphilis in Pregnant Women in Japan in 2016 and 2022

**DOI:** 10.7759/cureus.56292

**Published:** 2024-03-16

**Authors:** Eijiro Hayata, Shunji Suzuki, Shin-ichi Hoshi, Akihiko Sekizawa, Yoko Sagara, Isamu Ishiwata, Tadaichi Kitamura

**Affiliations:** 1 Division of Maternal and Child Health, Japan Association of Obstetricians and Gynecologists, Tokyo, JPN; 2 Infectious Diseases, Japanese Foundation for Sexual Health Medicine, Tokyo, JPN

**Keywords:** japan, penicillin g, congenital syphilis, pregnancy, syphilis

## Abstract

Since 2012, the rate of syphilis infection has increased dramatically in high-income countries including Japan. In this study, we examined the rate of syphilis infection among pregnant women and perinatal outcomes in the syphilis-infected pregnancy in 2022 in Japan, and compared the results with those in 2016. We requested 2,005 obstetric institutes to provide information on syphilis infection in pregnant women who delivered in 2022. A total of 1,346 obstetrical facilities responded with valid information. We compared the results with those in our previous study. The prevalence of syphilis-infected pregnant women was 1/1,215. The incidence of preterm delivery, intrauterine fetal demise and congenital syphilis in surviving neonates in the syphilis-infected pregnancy were 9%, 2% and 7%, respectively. The prevalence of syphilis-infected pregnant women has increased significantly, while the incidence of congenital syphilis seems to have decreased clinically.

## Introduction

Syphilis is one of the serious sexually transmitted infections (STIs) due to the bacterium Treponema pallidum (TP). Since 2012, the incidence of syphilis infection has risen dramatically in high-income countries around the world including Japan [1-3]. In Japan, among men the peak range in age has been observed from their 20s to 50s, while among women the trend has continued to be common among those in their 20s and 30s [3]. It has been recognized that if a pregnant woman has syphilis, it may infect her fetus during pregnancy or labor, resulting in congenital syphilis [1,2,4]. Therefore, the diagnosis of mother-to-child transmission of syphilis by blood tests on newborns even if there are no clinical symptoms of syphilis has been recommended by the experts in the World Health Organization [5].

In Japan, all pregnant women have been routinely screened for syphilis infection with TP hemagglutination (TPHA) and rapid plasma reagin (RPR) test at the first prenatal check-up by the Japanese public funds [6]. In addition, the obstetrical guidelines by the Japan Society of Obstetrics and Gynecology and Japan Association of Obstetricians and Gynecologists (JAOG) recommend that all pregnant women who are not tested in their first prenatal check-up should be tested for syphilis in subsequent visits [6].

In an earlier study, we observed the status of syphilis-infected pregnancy in Japanese women who gave birth at ≥ 22 weeks of gestation in 2016 in Japan [7]. In the study, we requested 2,458 obstetrical facilities that are members of the JAOG to provide information on syphilis screening tests and 1919 (78.1%) of them responded. Considering the response rate and the rate of implementation of confirmation tests, the number of syphilis-infected pregnant Japanese women was estimated to be 250 (1/4,022) in 2016 in Japan.

In this study, we evaluated the prevalence of syphilis infection in pregnant women in 2022 in Japan and the perinatal outcomes of the pregnancies with syphilis infection and compared the results of the current survey with those in 2016.

## Materials and methods

The protocol of this study was approved by the Ethics Committee of the JAOG (202308_2).

In October 2023, we requested 2,005 obstetric institutes that are members of the JAOG to provide information concerning syphilis infection in pregnant women who gave birth at ≥ 22 weeks of gestation between January 1 and December 31, 2022. It was an internet-based self-administered questionnaire survey, and the survey respondents were the directors of the department of obstetrics (physicians). ln the survey, all questions were optional questions, and responses that were answered in full and showed no discrepancies in content were considered as valid information. Syphilis infection in pregnant women was diagnosed when both RPR and TP antibodies were positive, and the women had clinical symptoms or when the RPR was ≥ 16. Congenital syphilis was diagnosed using blood or other samples taken from infants. A total of 1,346 (67.1%) out of the 2,005 institutes responded with valid information on 455,696 women which accounted for approximately 59% of the total number of deliveries in Japan during the study period in Japan (annual deliveries in Japan in 2022: approximately 770,000).

The additional surveys other than those about the prevalence of syphilis infection were as follows: maternal age, timing of the diagnosis of syphilis and perinatal outcomes such as the rates of preterm delivery, intrauterine fetal demise and congenital syphilis diagnosed by blood tests on newborns. As for the timing of the diagnosis, the question was asked in which trimester of the pregnancy. For pregnant women diagnosed with syphilis during the second or third trimester of pregnancy, the results of syphilis tests during the first trimester of pregnancy were verified. In addition, we compared these data with those in our previous study [6] to examine the recent trends, because in Japan the clinical introduction of penicillin G for intramuscular injection was re-approved in January 2022 [8]. We understand the importance of the phase of syphilis concerning the occurrence of congenital syphilis. However, in the previous study in 2016 [6] the stage of syphilis could not be determined because there were no symptoms of infection and the timing of infection was unknown in most cases of syphilis diagnosed during pregnancy. Therefore, in this study we did not request the information about the phase of syphilis.

Data are presented as numbers (percentage, %). For categorical variables, the Χ2 or Fisher’s exact test was performed with the statistical software SAS version 8.02 (SAS Institute, Cary, NC, USA). Differences with p < 0.05 were considered significant.

## Results

Results of the current survey

Table 1 shows the prevalence of syphilis infection during pregnancy in Japan by maternal age. The total prevalence of syphilis infection in the pregnant women was 1/1,215. The incidence of syphilis infection during pregnancy was higher among younger women (p < 0.01).

**Table 1:** Distribution of syphilis-infected pregnant women who delivered at ≥ 22 weeks of gestation by maternal age in 2022 in Japan. Data are presented as number or rate. *P < 0.01 vs. groups of maternal age 20-29, 30-39 and ≥ 40 years. #P < 0.01 vs. maternal age 20-29 years.

Maternal age (y)	Total number	Number of syphilis-infected women	syphilis-infection rate
≤ 19	3,504	18	1/195*
20-29	139,432	237	1/588#
30-39	245,730	108	1/2,275
≥ 40	28,014	8	1/3,502
Unknown	39,016	4	1/9,754
Total	455,696	375	1/1,215

Table 2 shows the timing of the diagnosis of syphilis infection during pregnancy in Japan. Of the 375 syphilis-infected women during pregnancy, 303 (81%) were diagnosed during the first trimester of pregnancy, while 72 (19%) were diagnosed after the second trimester. In 18 of the latter, the results of syphilis tests during the first trimester were negative (5% of all syphilis-infected pregnant women).

**Table 2:** Timing of diagnosis of syphilis in the pregnant women who delivered at ≥ 22 weeks of gestation by maternal age in 2022 in Japan. Data are presented as number (percentage).

Maternal age (y)	Total number	Diagnosis during the first trimester	Diagnosis during the second or third trimester/ Result of the test during the first trimester: negative	Diagnosis during the second or third trimester/ Result of the test during the first trimester: unexecuted
≤ 19	18	15 (83%)	0 (0%)	3 (17%)
20-29	237	191 (81%)	13 (5%)	33 (14%)
30-39	108	90 (83%)	4 (4%)	14 (13%)
≥ 40	8	5 (63%)	0 (0%)	3 (37%)
Unknown	4	2 (50%)	1 (25%)	1 (25%)
Total	375	303 (81%)	18 (5%)	54 (14%)

Table 3 shows the perinatal outcomes of the syphilis-infected pregnant women in Japan. The incidence of preterm delivery, intrauterine fetal demise and congenital syphilis in surviving neonates in the syphilis-infected pregnancy were 9%, 2% and 7%, respectively.

**Table 3:** Perinatal outcomes at ≥ 22 weeks of gestation of the syphilis-infected women by maternal age in 2022 in Japan. Data are presented as number (percentage). *Congenital syphilis in surviving neonates.

Maternal age (y)	Total number	Premature delivery	Intrauterine fetal demise	Congenital syphilis*
≤ 19	18	1 (6%)	0 (0%)	1 (6%)
20-29	237	20 (8%)	8 (3%)	18 (8%)
30-39	108	8 (7%)	0 (0%)	9 (8%)
≥ 40	8	4 (50%)	1 (13%)	0 (0%)
Unknown	4	0 (0%)	0 (0%)	0 (0%)
Total	375	33 (9%)	9 (2%)	28 (7%)

Comparison with previous survey results

Table 4 shows the comparison of the results between 2016 and 2022. Compared to our previous study concerning syphilis infection during pregnancy in 2016 [7], the prevalence of syphilis infection during pregnancy increased about 3.3 times (p < 0.01) as shown in Table 4. The significant increase was found in all age groups, while the prevalence of pregnant women whose results of syphilis testing during the first trimester were negative or whose first syphilis test was performed beyond the second trimester has not changed.

**Table 4:** Comparison of results between 2016 and 2022 The data in 2016 are taken from Ref. 7. Data are presented as rate and percentage. *P < 0.05 vs. 2026.

Year	2016	2022
Syphilis-infection rate by maternal by maternal age: total	1/4,022	1/1,215*
< 20 years	1/537	1/195*
20-29 years	1/2,449	1/588*
30-39 years	1/8,091	1/2,275*
≥ 40 years	1/6,012	1/3,502*
Diagnosis during the first trimester	78%	81%
Diagnosis during the second or third trimester/ Result of the test during the first trimester: negative	5%	5%
Diagnosis during the second or third trimester/ Result of the test during the first trimester: unexecuted	16%	14%
Premature delivery	8%	9%
Intrauterine fetal demise/early neonatal death	4%	2%
Congenital syphilis	14%	7%

The incidence of preterm delivery did not change from 2016 to 2022. The decreases in the incidence of perinatal mortality and congenital syphilis did not reach statistical significance (p = 0.43 and 0.07, respectively).

## Discussion

A comparison of the survey results in 2016 and 2022 can be summarized as follows: in Japan the prevalence of syphilis-infected pregnancy increased significantly from 2016 to 2022, however, the incidence of congenital syphilis decreased clinically although it did not reach statistical significance. In recent years, the number of syphilis infections has increased dramatically in Japan as well as other high-income countries [1-3,9,10]. The number of syphilis infections has been increasing especially among young women, and the number of congenital syphilis has been also on the rise [9,10]. The current study is in line with these previous observations [1-3,9,10].

In this study, the timing of the diagnosis of syphilis during pregnancy did not change between 2016 and 2022, because the timing has been routinely offered during the first trimester of pregnancy. After the previous study [7], awareness activities were raised about the need for early prenatal check-up and the prevention of STIs during pregnancy in the JAOG; however, unfortunately the current results did not indicate the effectiveness of the activities. In addition, while previous awareness activities such as STI prevention had mainly targeted young age women [7,11,12], the increase in syphilis infection among all age groups in this study may suggest that the target of the awareness activities should be expanded. It is important to educate all pregnant women about the increasing prevalence of syphilis infection and prevention of the infection during pregnancy at prenatal check-ups, and if there is even the slightest suspicion of syphilis infection, it is important to actively conduct re-examination of syphilis.

In this study, the difference in the perinatal outcomes of syphilis-infected pregnancy seemed to be present between 2016 and 2022 although they did not reach statistical significance. Although the number of syphilis-infected pregnant women has increased, congenital syphilis might be decreased due to early diagnosis and treatment, as obstetricians have become more aware of the disease. On the other hand, preterm deliveries and intrauterine fetal demise occur even when syphilis is not present, so there might not have been as much change as that of congenital syphilis. In addition, in Japan oral amoxicillin (OA) was commonly used to treat syphilis until 2021. However, it has been suggested that OA may not be effective in preventing congenital syphilis in pregnant women with late-stage syphilis [10,13]. In addition, OA requiring a median treatment time of 12 weeks has been indicated as a significantly longer time period for adequate treatment of syphilis during pregnancy before delivery [8]. The possibility that long-term medication may lead to poor adherence cannot be ruled out, especially in the absence of symptoms of syphilis [14]. In Japan, the clinical introduction of penicillin G for intramuscular injection was re-approved in January 2022 [8]. Therefore, a change in treatment approach is expected in Japan. However, another survey conducted during the same period showed that Japanese obstetricians and gynecologists had only about 14% experience with the use of intramuscular penicillin G in 2022 [15]. Therefore, a future examination considering the therapeutic effect of the parenteral penicillin G will be warranted based on the expanding awareness of the use of intramuscular penicillin G. We hope that the addition of the treatment method completed in a shorter time will further reduce the incidence of congenital syphilis, because the incidence of adverse outcomes such as congenital syphilis is expected to decrease not only clinically but also statistically significantly within a few years in Japan [8].

The data of this study were taken from almost all obstetric institutes in Japan; however, we understand the presence of several limitations in this study. First, this was a simple questionnaire survey only for obstetricians, not neonatologists. Therefore, the response seemed to be unclear for some questions, especially in the diagnosis of congenital syphilis. In addition, because the incidence of congenital syphilis is extremely rare, a huge number of subjects will be needed to achieve statistical significance. Second, this retrospective survey may have introduced some bias of information including some missing variables such as maternal age. In addition, data on adherence of pregnant women to the long-term treatment could not be evaluated.

## Conclusions

The prevalence of syphilis-infected pregnant women increased significantly in 2022 compared with that in 2016 in Japan; however congenital infection of syphilis did not increase, but rather showed a decreasing trend.
